# Mimicking myocardial infarction: a subarachnoid haemorrhage case report

**DOI:** 10.1093/omcr/omae154

**Published:** 2024-12-28

**Authors:** Ezzat Mohammed Hussain Aziz, Ahmed Qasim Mohammed Alhatemi, Hashim Talib Hashim, Amir Salam Khaleel

**Affiliations:** Coronary Care Unit, Al Nasiriyah Heart Hospital, Thi Qar 64001, Iraq; Department of Internal Medicine, Al Nasiriyah Teaching Hospital, Thi Qar 64001, Iraq; College of Medicine, Warith Al Anbiyaa University, Karbala 56001, Iraq; Coronary Care Unit, Al Nasiriyah Heart Hospital, Thi Qar 64001, Iraq

**Keywords:** neurology, cardiology, diagnostic testing

## Abstract

We present a case detailing the diagnostic challenges of a 23-year-old male presenting with a sudden severe headache, nausea, vomiting, and chest heaviness. Initial evaluation showed elevated blood pressure and respiratory rate. An emergency electrocardiogram (ECG) indicated ST-segment elevation myocardial infarction (STEMI), leading to immediate referral for percutaneous coronary intervention, which revealed normal coronary arteries. Further investigations identified a cisternal subarachnoid haemorrhage (SAH) on CT brain imaging. Despite multidisciplinary management, the patient’s condition rapidly deteriorated, resulting in cardiac arrest and mortality. Our case highlights the importance of thorough evaluation and multidisciplinary collaboration in managing complex presentations, emphasizing vigilance in recognizing and managing concurrent pathologies in young patients with acute symptoms.

## Introduction

The electrocardiogram is a vital tool for measuring cardiac electrical activity and diagnosing various heart conditions. One of the significant indicators on an ECG is the ST segment, which represents the interval between ventricular depolarization and repolarization. Common causes of ST segment elevation include acute myocardial infarction, pericarditis, and early repolarization. However, ST segment elevation (STE) can also occur in the context of non-cardiac conditions such as intracranial haemorrhage (ICH) [1].

ST-segment elevation in the context of intracranial haemorrhage presents a compelling yet intricate clinical scenario, where neurological and cardiovascular manifestations intersect. While traditionally associated with cardiac ischemia, STE in the setting of ICH raises challenging diagnostic and management considerations. Intracranial bleeding, whether traumatic or spontaneous, can elicit sympathetic surges, impacting cardiac repolarization and potentially inducing STE on electrocardiograms [1, 2].

The pathophysiology of ST elevation in ICH is multifactorial. Increased intracranial pressure and activation of the sympathetic nervous system can lead to catecholamine release, influencing cardiac dynamics. This neurological insult may provoke myocardial injury, with ensuing ECG changes resembling those seen in acute coronary syndromes. However, distinguishing primary cardiac events from neurologically induced ECG alterations involves assessing clinical symptoms, troponin levels, and imaging studies to accurately diagnose the underlying cause of ST segment elevation, which is pivotal for appropriate clinical management [3].

The elevation of ST-segments in ICH may not always correlate with coronary artery disease. Instead, it reflects the intricate neurocardiac interaction, emphasizing the significance of interdisciplinary collaboration between neurologists and cardiologists. Neurogenic stunned myocardium (NSM) is a recognized entity, characterized by transient cardiac dysfunction secondary to neurological insult, contributing to ST-segment elevation [4].

The clinical implications of STE in ICH not only pose a diagnostic challenge but are also associated with heightened morbidity and mortality, emphasizing the need for a comprehensive understanding of the interplay between neurological and cardiovascular dynamics [5].

This case report of rare ST-segment elevation in ICH highlights the importance of understanding the connection between intracranial haemorrhage and cardiac issues. It emphasize the need for a careful approach in identifying and treating this complex condition, recognizing how neurological and cardiac problems are related in acute intracranial events. This understanding is crucial for improving patient care and outcomes when ST-segment elevation complicates intracranial haemorrhage.

## Case report

A 23-year-old male patient presented to the emergency department with a sudden onset of severe frontal headache lasting for 2 h. He experienced associated symptoms of nausea, vomiting, and chest heaviness. He has a unremarkable medical record and denies the use of illicit drugs. However, he is a smoker with a history of 23 pack-years but does not consume alcohol.

On physical examination, the young male appeared distressed but was fully conscious and oriented to time, place, and person. Chest auscultation revealed normal vesicular breathing sounds, while cardiovascular and abdominal examinations were inconclusive. Neurological examinations demonstrated neck stiffness, dilated pupils reactive to light, normal plantar reflexes, and no focal neurological deficits.

His vital signs were as follows: blood pressure 178/103 mmHg, respiratory rate 26 breaths/min, temperature 38.9°C, heart rate 87 beats/min, and oxygen saturation of 94%.

**Figure 1:**
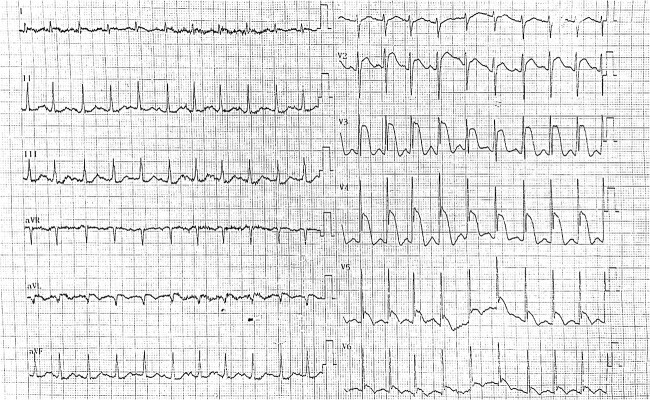
Electrocardiogram (ECG) obtained in the emergency department demonstrating significant ST-segment elevation (>2 mm) in leads V2-V5, suggestive of anterior myocardial infarction.

Emergency tests were initiated. An ECG revealed ST segment elevation >2 mm in leads V2-V5, consistent with STEMI as the top of our differential diagnosis, requiring confirmation by cardiac markers (Fig. 1). With prompt referral to a tertiary cardiac centre implemented, the patient received a 300 mg aspirin load while being transferred to the catheter lab. Troponin levels were significantly elevated at 1.48 mg/dl (normal <0.16 mg/dl).

**Figure 2:**
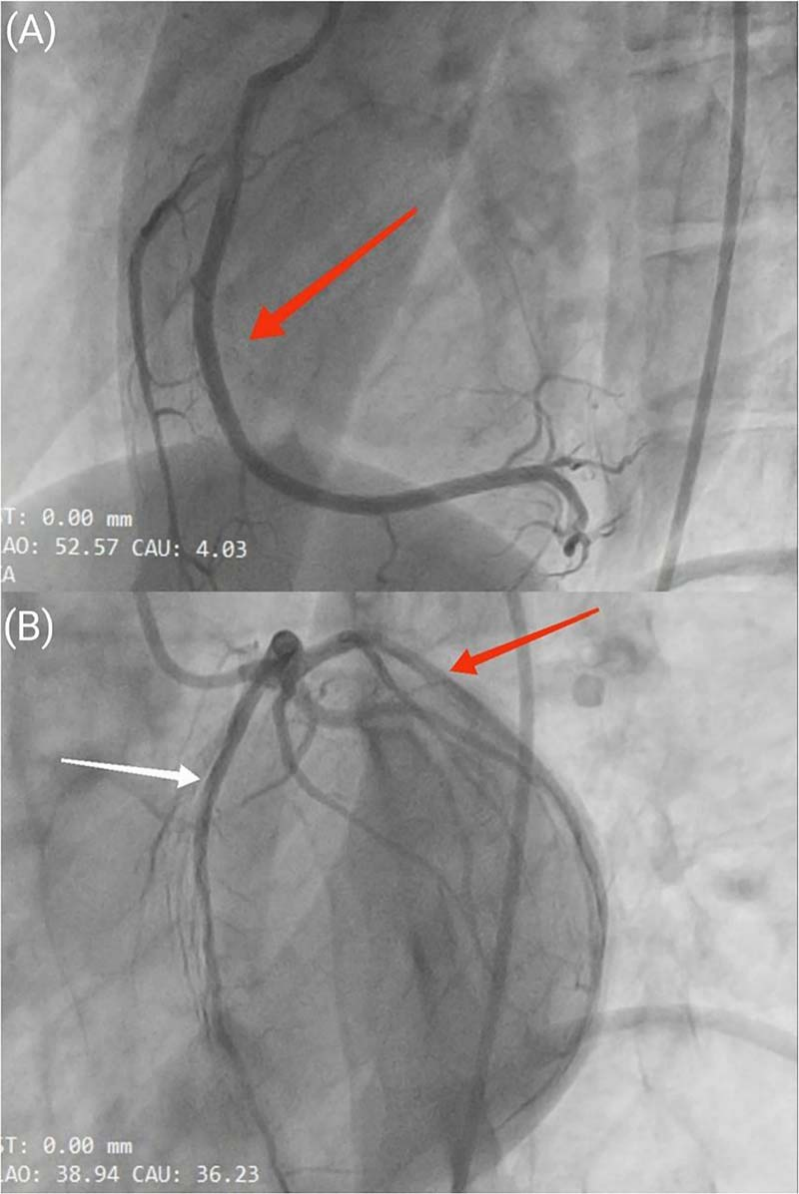
(**A**) Coronary angiography in the left anterior oblique (LAO) view, illustrating a normal right coronary artery (RCA) as indicated by the red arrow. (**B**) Coronary angiography in the LAO caudal view, displaying normal left circumflex artery (LCX) (red arrow) and left anterior descending artery (LAD) (white arrow).

Percutaneous coronary intervention was performed via the femoral artery, and the result showed normal coronary arteries with thrombolysis in myocardial infarction (TIMI) flow grade of 3. (Fig. 2A and B).

Video 1: Showing normal right coronary artery (RCA) and Video 2: Showing normal left anterior descending artery (LAD) and left circumflex artery (LCX).

**Figure 3:**
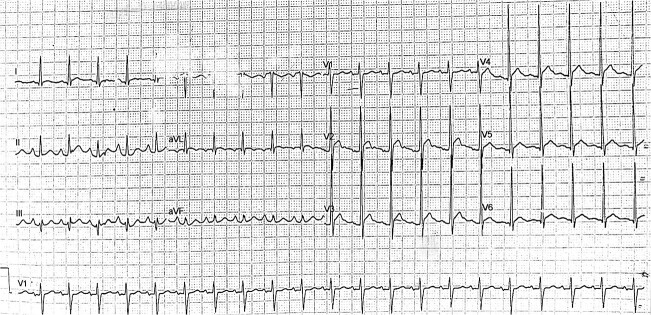
Post-angiography ECG showing a normal sinus rhythm accompanied by left ventricular hypertrophy (LVH).

His ECG after coronary angiography revealed normal sinus rhythm with left ventricular hypertrophy LVH (Fig. 3). An echocardiogram was performed, revealing normal ventricular function with no regional wall motion abnormalities (RWMA).

Following coronary intervention, he was admitted to the medical ward for further assessment and investigation. Blood samples were drawn for a complete blood count, random blood sugar, renal function tests, and CRP. The results revealed lymphocytosis and mildly elevated CRP. (Table 1).

**Table 1:** Blood tests showing lymphocytosis and mild elevated CRP

**Test**	**Results**	**Normal value**
HB	14.9	12–16 g/dl
WBC	17.9	4–10 10^3^/microliter
PLT	123	150–350 10^3^/microliter
B. Urea	39	10–45 mg/dl
Creatinine	0.8	0.4–1.1 mg/dl
CRP	12	<10 mg/L
R.B.S	107	140–200 mg/dl

**Figure 4:**
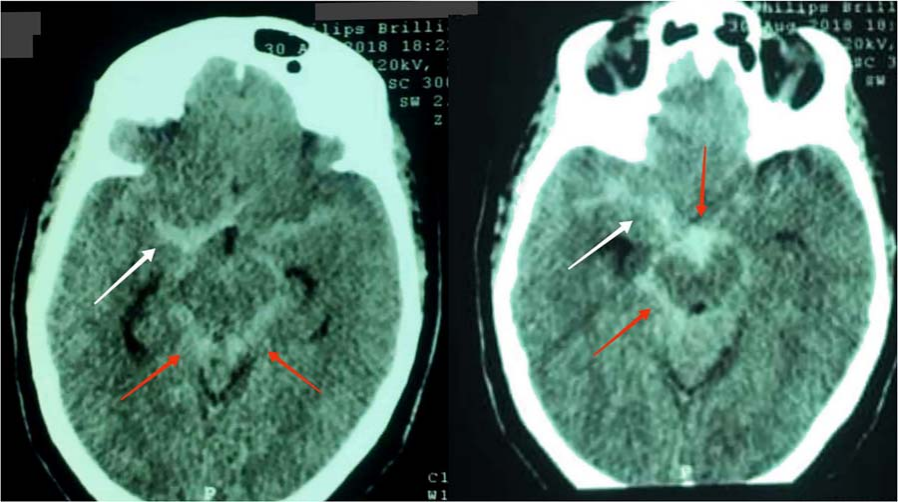
Brain CT axial view showing cisternal SAH (red arrows) with extension anterior to the right temporal lobe (white arrows).

We proceeded further with CT brain to exclude serious cause of headache (Fig. 4). His brain CT showed cisternal subarachnoid haemorrhage SAH with extension anterior to the right temporal lobe. Abdominal ultrasound screening was performed to rule out polycystic kidney disease which was negative and cerebral CT angiography was scheduled to exclude cerebral aneurysm Nimodipine 60 mg every 4 h was initiated, with a target blood pressure of 160/100 mmHg.

On the second day, his condition suddenly deteriorated, culminating with cardiac arrest. Therefore, cardiopulmonary resuscitation (CPR), resulting in a Glasgow Coma Scale score (GCS) of 6. The patient was subsequently, intubated and placed on mechanical ventilation in the Intensive Care Unit (ICU). Due to his unstable condition in the ICU, we could not perform a repeated CT brain scan or the planned cerebral CT angiography.

Over the next 7 days, we diligently monitored him with a strict multidisciplinary team. A nasogastric tube was inserted for feeding and fluid replacement. His medications included intravenous fluids, antibiotics, proton pump inhibitors, and nimodipine.

On the 8th day, he suddenly developed ventricular fibrillation, and despite CPR with more than five defibrillations, we were unable to revive him and death was the final outcome.

Table 2 presents a series of reported instances where subarachnoid haemorrhage mimicked ST-segment elevation myocardial infarction, emphasizing the diagnostic complexity and varied clinical presentations associated with these cases. The patients’ ages ranged from 23 to 68 years, illustrating that this phenomenon can affect a wide demographic.

**Table 2:** Summary of reported cases of subarachnoid haemorrhage mimicking acute myocardial infarction

**Case/Study**	**Age of the patient**	**Clinical presentation**	**Diagnostic modalities**	**Treatment**	**Final outcome**
Park I, Kim YJ, Ahn S, Sohn CH, Seo DW, Kim WY [6].	48	Cardiac arrest	ECG, Echocardiography, brain CT scan, coronary angiography	Vasopressors	Mortality
Chen HY [7].	66	Transient conscious loss	ECG, Echocardiography, brain CT scan, coronary angiography	Conservative treatment	Mortality
Abdalla MS, Smith BC, Kirchner A, Abu Nseir M, Mokhtar M, Abdulrahman A, Saad E [8].	68	Neck, left shoulder, and upper back pain	ECG, Echocardiography, brain CT scan, CT pulmonary angiography	Observation	Recovery
Enache I, Radu RA, Terecoasă EO, Dorobăţ B, Tiu C [9].	60	Sudden loss of consciousness	ECG, Echocardiography, brain CT scan, coronary angiography	Endovascular coiling	Recovery
Mortazavi A, Jelodar S, Edraki K, Narimani S, Ghorbani M, Karimi-Yarandi K, Asaadi S [10].	47	Severe headache	ECG, brain CT scan,coronary angiography	Endovascular closure of brain aneurysm	Recovery
Lin XQ, Zheng LR [11].	37	Sudden dizziness and left limb weakness	ECG, Echocardiography, brain CT scan, chest X-ray coronary angiography	Symptomatic treatment with vasodilators	Recovery

Common clinical presentations included severe headache, sudden loss of consciousness, and chest pain, with ECG changes showing ST-segment elevation. Diagnostic modalities often involved a combination of ECG, echocardiography, brain CT scan, and coronary angiography to differentiate between primary cardiac events and neurologically induced ECG changes.

Treatment strategies varied, with some patients receiving conservative management and others undergoing more invasive procedures like endovascular coiling. The outcomes also varied, with some cases resulting in mortality and others in improvement, underscoring the critical importance of prompt and accurate diagnosis, interdisciplinary collaboration, and tailored treatment approaches in managing these complex cases. The literature highlights the significant overlap between neurological and cardiovascular symptoms in SAH, necessitating a high index of suspicion and comprehensive evaluation to optimize patient outcomes.

## Discussion

ST-segment elevation in intracranial haemorrhage presents a complex and often perplexing clinical scenario. While the electrocardiographic changes are not exclusive to ICH, they signify a critical interplay between neurological and cardiovascular systems [12].

The elevation of ST-segments can occur through various mechanisms, including heightened sympathetic activity, catecholamine release, or direct cardiac injury due to increased intracranial pressure [13, 14].

However, it’s essential to distinguish this phenomenon from primary myocardial infarction by the presence of reciprocal changes in the latter, necessitating a comprehensive clinical evaluation [4].

Managing ST-segment elevation in the context of ICH involves a tailored approach. Rigorous neurological assessment is crucial, directing interventions based on the primary cerebral pathology [15]. Hemodynamic stability should be prioritized, balancing the need to control blood pressure to mitigate further cerebral injury while ensuring adequate perfusion of the myocardium. Antithrombotic therapy requires careful consideration, weighing the risks of expansion of the intracranial bleed against the risks of acute coronary occlusion. In select cases, an invasive strategy with coronary angiography may be necessary to definitively diagnose and manage the coronary artery disease, ideally with neurointerventionalist availability due to the elevated risk of ICH [15].

Concurrently, attention to cardiac monitoring and potential complications, such as arrhythmias or myocardial infarction, is imperative [6]. Advanced imaging techniques, such as brain imaging and echocardiography, play a pivotal role in unraveling the intricate relationship between the brain and the heart in these [6].

Timely and accurate diagnosis ensures appropriate management, addressing both the neurological and cardiac aspects of the condition [6]. A nuanced understanding of neurocardiac interactions is paramount for effective clinical decision-making, highlighting the necessity for a collaborative and multidisciplinary approach to optimize patient care in these intricate cases [16].

## Conclusion

This case highlights the diagnostic complexity encountered when symptoms suggestive of myocardial infarction lead to the discovery of a cisternal subarachnoid haemorrhage. The scenario emphasize the critical interplay between neurological and cardiovascular manifestations. Effective management in such cases necessitates a multidisciplinary approach involving neurology, cardiology, and critical care specialties. Collaborative efforts are essential for accurate diagnosis, timely intervention, and improved patient outcomes.

## Supplementary Material

Videos_omae154
