# Supplementary material for: Mimicking myocardial infarction: a subarachnoid haemorrhage case report
Source: Oxf Med Case Reports. 2024 Dec 28;2024(12):omae154. doi: 10.1093/omcr/omae154 (PMC11682489; doi:10.1093/omcr/omae154)
Supplement: Videos_omae154 [file videos_omae154.pptx]

## Slide 1
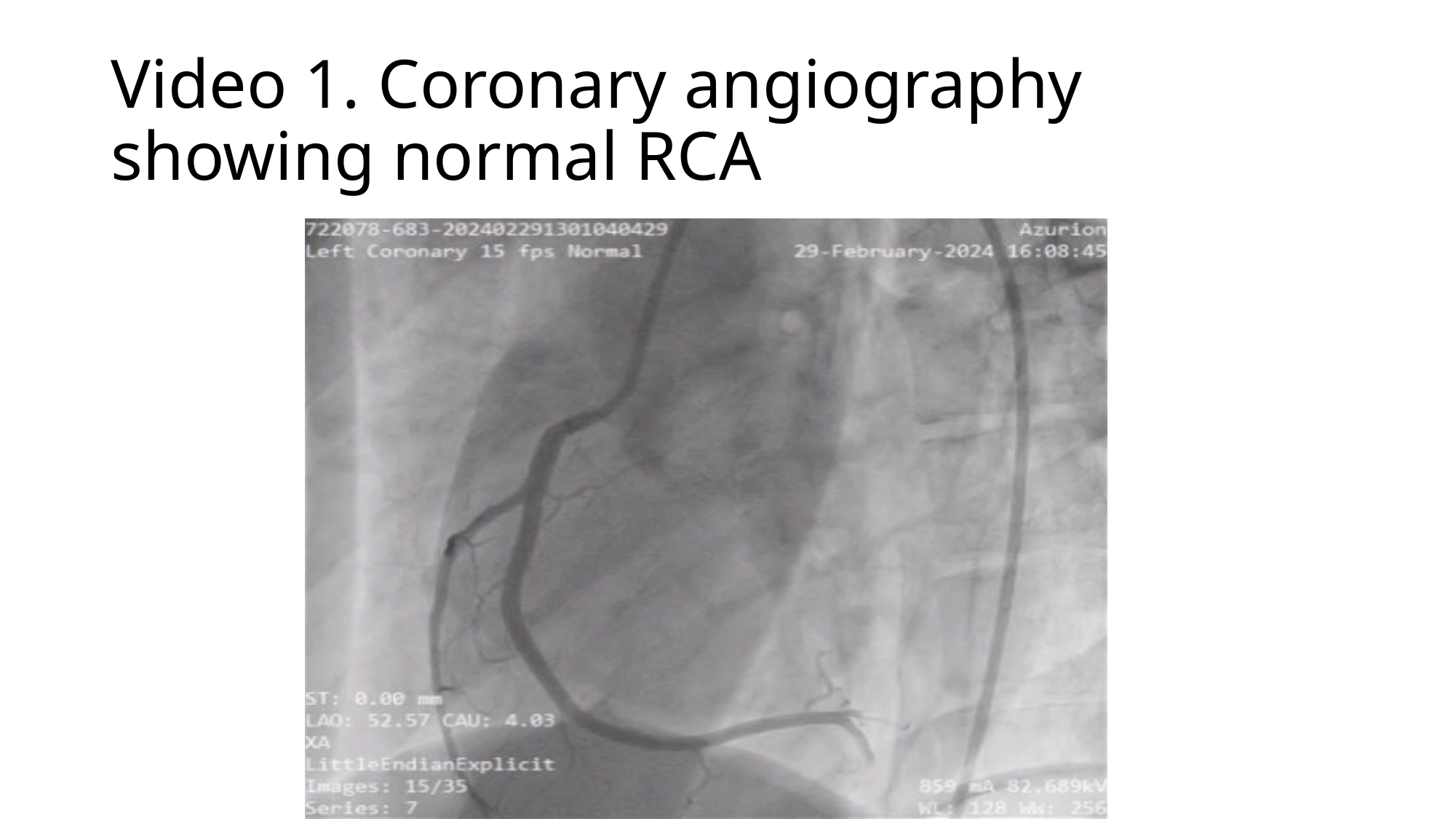

# Video 1. Coronary angiography showing normal RCA

## Slide 2
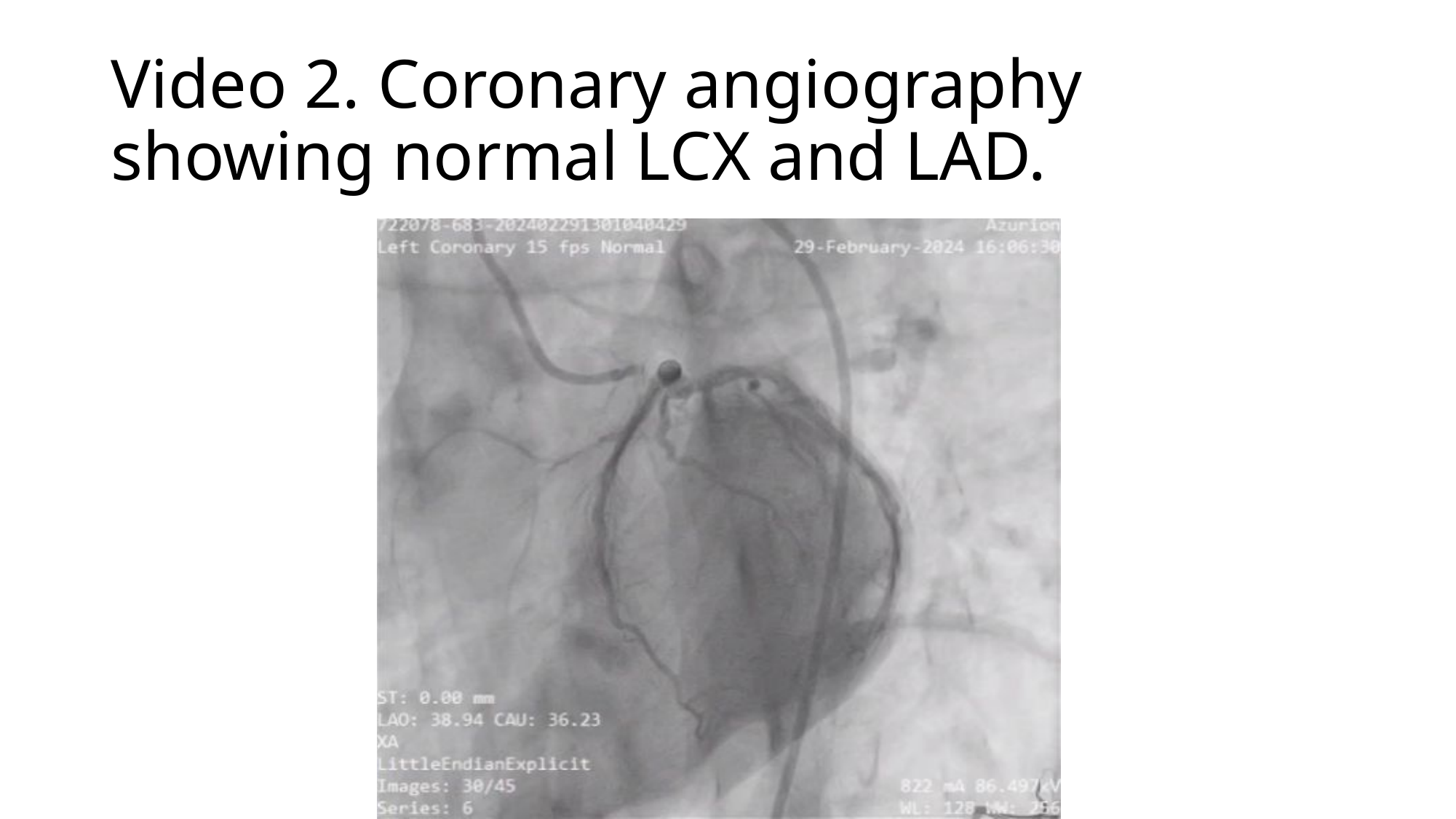

# Video 2. Coronary angiography showing normal LCX and LAD.
